# TMAO as a potential biomarker and therapeutic target for chronic kidney disease: A review

**DOI:** 10.3389/fphar.2022.929262

**Published:** 2022-08-12

**Authors:** Ye Zixin, Chen Lulu, Zeng Xiangchang, Fang Qing, Zheng Binjie, Luo Chunyang, Rao Tai, Ouyang Dongsheng

**Affiliations:** ^1^ Department of Clinical Pharmacology, Xiangya Hospital, Central South University, Changsha, China; ^2^ Hunan Key Laboratory of Pharmacogenetics, Institute of Clinical Pharmacology, Central South University, Changsha, China; ^3^ Engineering Research Center of Applied Technology of Pharmacogenomics, Ministry of Education, Changsha, China; ^4^ National Clinical Research Center for Geriatric Disorders, Changsha, China; ^5^ Hunan Key Laboratory for Bioanalysis of Complex Matrix Samples, Changsha, China; ^6^ Department of Clinical Pharmacy, Affiliated Hospital of Xiangnan University, Chenzhou, China

**Keywords:** trimethylamine N-oxide, chronic kidney disease, gut microbiota, targeted TMAO drugs, mechanism, treatment

## Abstract

The gut microbiota and its metabolites have become a hotspot of recent research. Trimethylamine N-oxide (TMAO) metabolized by the gut microbiota is closely related to many diseases such as cardiovascular disease, chronic kidney disease, type 2 diabetes, etc. Chronic kidney disease (CKD) is an important contributor to morbidity and mortality from non-communicable diseases. Recently, increasing focus has been put on the role of TMAO in the development and progress of chronic kidney disease. The level of TMAO in patients with chronic kidney disease is significantly increased, and a high level of TMAO deteriorates chronic kidney disease. This article describes the relationship between TMAO and chronic kidney disease and the research progress of drugs targeted TMAO, providing a reference for the development of anti-chronic kidney disease drugs targeted TMAO.

## 1 Introduction

Chronic kidney disease (CKD) refers to abnormal kidney structure and function caused by various reasons such as diabetes, hypertension, and older age. A persistently reduced glomerular filtration rate (GFR < 60 ml/min/1.73 m^2^ for more than 3 months) is the specific diagnostic criterion for CKD (National Kidney, 2002; Stevens and Levin, 2013). The global incidence of CKD in 2017 was 9.1%, and approximately 1.2 million people died of CKD. The prevalence of CKD is about 9.5%, and the death toll is about 176,000 in China in 2017 (Collaboration, 2020). It is estimated that CKD will become the fifth leading cause of early death by 2040 (Foreman et al., 2018). CKD is not only an important contributor to morbidity and mortality from non-communicable diseases, but also an important risk factor for cardiovascular disease (Collaboration, 2020).

Early-stage CKD is asymptomatic and difficultly diagnosable. However, slowing early-stage CKD progression provides economic benefits (Trivedi et al., 2002) and prevents the development of End-Stage Kidney Disease (ESKD) and cardiovascular complications (Group, 2013). The mechanisms of CKD have not been fully elucidated. Nephron loss, nephron hypertrophy, impaired glomerular filtration, and fibrosis are summarized as primary pathophysiological characteristics of CKD (Romagnani et al., 2017). The first-line drugs are inhibitors of the renin-angiotensin system (RAS), including angiotensin converting enzyme (ACE) inhibitors and angiotensin II receptor blockers (ARBs), which have limited effectiveness and only delay CKD progression (Ruggenenti et al., 2012; Ruiz-Ortega et al., 2020). What’s more, Inhibitors of RAS must be used with caution in patients with advanced CKD (stages 4, 5), because they can lead to hyperkalemia, acute declines in estimated glomerular filtration rate (eGFR) (Leon and Tangri, 2019). Therefore, developing novel medicines preventing CKD generation and progression and even achieving restoration of renal function in all patients with CKD are of paramount importance. Clarifying the mechanisms of CKD is an important step to find valuable targets for CKD treatment.

In addition, Common CKD biomarkers such as Cystatin C, creatinine, and Proteinuria have limitations, especially the low sensitivity of serum creatinine and eGFR in early-stage CKD and the limited application of uric acid under certain circumstances (Chonchol et al., 2007; Fassett et al., 2011). Progression of CKD is irreversible and there are no effective therapies for advanced CKD. The earlier the diagnosis, the greater the patients will benefit. Therefore, it is necessary to explore new biomarkers for early diagnosis and prediction of CKD.

TMAO can be detected in plasma, urine and feces, there are various methods to detect, such as LC-MS, and molecularly imprinted polymer (MIP) based electrochemical sensor (Romano et al., 2015; Zhao et al., 2015; Lakshmi et al., 2021; Rox et al., 2021). These methods were rapid, high-throughput and cost-effective for analysis TMAO, and preparation of samples was simple. LC-MS was a common method to detect TMAO which was easy to operate in clinical application.

**FIGURE 1:**
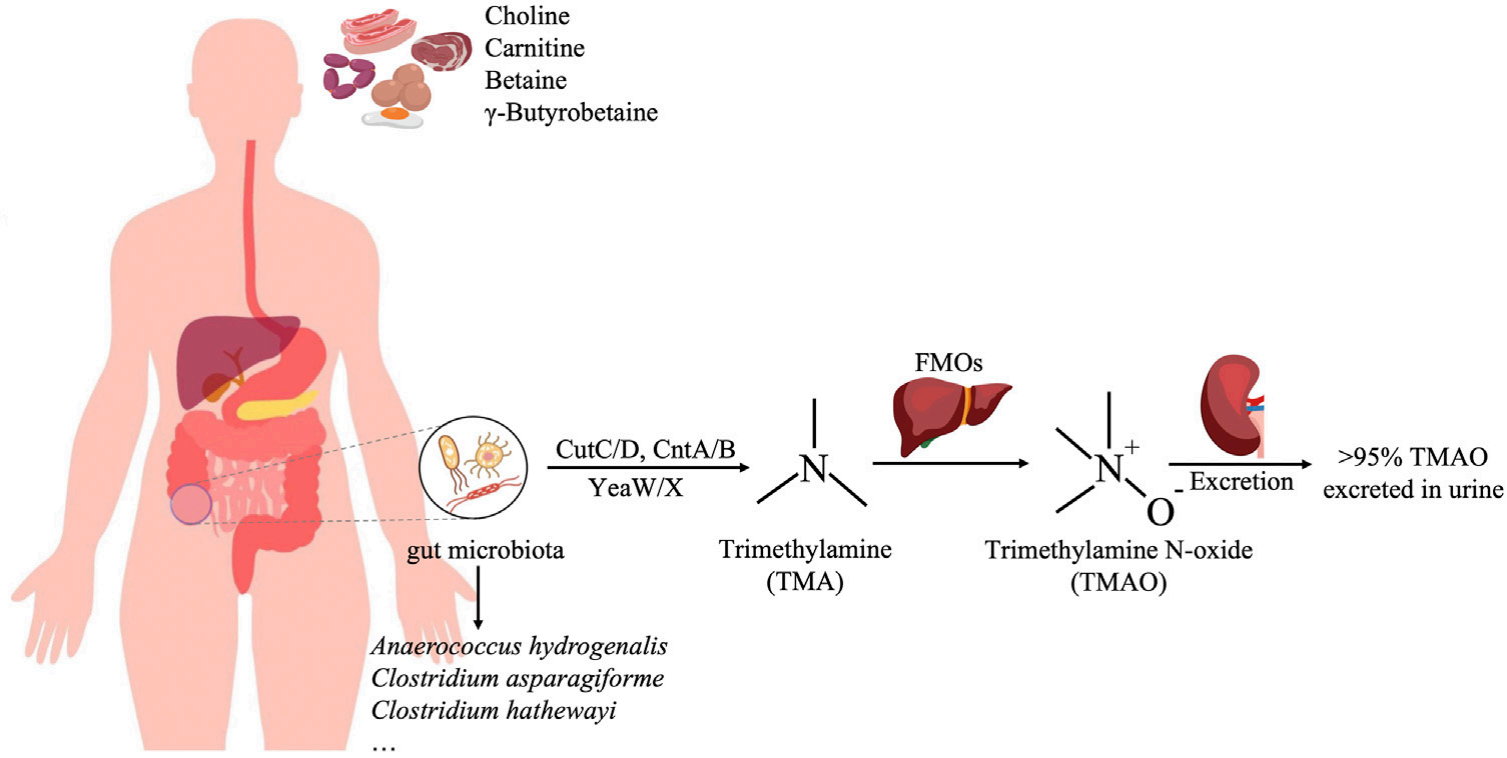
The production process of TMAO. Red meat, egg, and sausage are rich in choline and carnitine, which are metabolized by the gut flora, such as *Anaerococcus hydrogenalis*, *Clostridium asparagiforme*, and *Clostridium hathewayi*, into trimethylamine (TMA) in the colon, and then oxidized to TMAO in the liver by Flavin-containing monooxygenase 3 (FMO3).

Trimethylamine N-oxide (TMAO) is a metabolite of choline, L-carnitine, and betaine in the diet. Choline and L-carnitine that are not absorbed in the small intestine are metabolized by the gut flora into trimethylamine (TMA) in the colon and then oxidized to TMAO in the liver by Flavin-containing monooxygenase 3 (FMO3) (Bennett et al., 2013). The gut microbiota produced TMA is various and abundant, such as *Anaerococcus hydrogenalis*, *Clostridium asparagiforme*, *Clostridium hathewayi* (Romano et al., 2015)*.* The majority (greater than 95%) of trimethylamine N-oxide was excreted in urine, only 4% of the dose was excreted in the feces and <1% in the breath (Figure 1) (Al-Waiz et al., 1987). Studies have shown that TMAO is closely related to a variety of diseases, such as cardiovascular disease, kidney disease, type 2 diabetes, and tumors. In recent years, the relationship between TMAO and CKD has been gradually recognized. TMAO level is related to the occurrence and prognosis of CKD, and it can serve as a potential risk factor for the development of CKD (Cho and Caudill, 2017). At present, the treatment of CKD mainly focuses on controlling its progression rate and related complications, and the treatment options are very limited. TMAO is expected to become a new target for CKD treatment and provide new options for CKD treatment (Tomlinson and Wheeler, 2017). This article reviews the relationship between TMAO and chronic kidney disease and the research progress of targeted TMAO drugs in order to provide a theoretical reference for the development of new anti-chronic kidney disease drugs.

## 2 Trimethylamine N-oxide and chronic kidney disease

### 2.1 Trimethylamine N-oxide is a risk factor for chronic kidney disease

Risk factors for chronic kidney disease are various, including disease factors (Diabetes, hypertension, autoimmune diseases, systemic infections, urinary tract infections, acute kidney injury, etc.), Sociodemographic factors (age, gender, ethnicity, and family history of CKD, etc.), and lifestyle factor (smoking, heavy alcohol use, obesity, etc.) (Taal and Brenner, 2008; Drawz and Rahman, 2015). eGFR, an important indicator for kidney function, is used for diagnosis of CKD and assessment of progression of CKD. The common biomarkers of eGFR are Cystatin C and creatinine. (Fassett et al., 2011; Webster et al., 2017). These biomarkers’ measured concentrations are easily affected by factors other than eGFR. What’s more, Proteinuria, as a measure of kidney damage, is associated with the progression of CKD (Inker et al., 2014). An additional limitation of proteinuria is that it’s associated with rapid progression but that is not always present in CKD or may vary in different CKD stages (Group, 2013). Serum creatinine, eGFR, and proteinuria are insensitive and reliance on these may delay the early diagnosis and treatment (Fassett et al., 2011). Potential biomarkers have tried to overcome the limitations of current clinical biomarkers, such as biomarkers for the early stages of CKD and diagnosis of CKD in elderly individuals in whom the current clinical biomarkers have limited sensitivity and specificity (Canadas-Garre et al., 2019). The search for new relevant biomarkers to better stratify patients with CKD according to the risk of progression, morbidity, and mortality is underway.

Metabolomic studies in CKD found TMAO is a potential biomarker that may help identify CKD patients (Canadas-Garre et al., 2019). The TMAO level of CKD patients is higher than that of the control (Tang et al., 2015), and TMAO has a strong association with CKD in a validation study, 80% sensitivity with other six urinary metabolites including glutamate, guanidoacetate, and α-phenylacetylglutamine (all increased in CKD), 5-oxoproline, taurine, and citrate (all decreased in CKD) (Posada-Ayala et al., 2014). The levels of TMA and TMAO before hemodialysis in patients with end-stage renal disease (ESRD) are also higher than those in the control (Bain et al., 2006). High circulating TMAO and baseline choline level have a strong correlation with the occurrence of CKD (Rhee et al., 2013). At the early stages of CKD, a high TMAO level in plasma indicates a lower estimated glomerular filtration rate (eGFR) (Tang et al., 2015; Xu et al., 2017). In a cross-sectional study, Heart failure with preserved ejection fraction (HFpEF) and renal function were closely related with plasma TMAO levels and TMAO may serve as a diagnostic biomarker for HFpEF and renal function (Guo et al., 2020).

The impaired renal TMAO clearance may lead to elevated circulating TMAO concentration and reduced glomerular filtration rate, which could aggravate burden of kidney (Toyohara et al., 2010; Hai et al., 2015). In another study, there was a strong correlation between plasma TMAO levels and mGFR in CKD patients, that can be mainly related to a decrease in TMAO glomerular filtration (Pelletier et al., 2019). In animal models, it was found that increasing TMAO would aggravate renal fibrosis and renal dysfunction (Tang et al., 2015).

High TMAO concentration is associated with long-term mortality in patients with CKD and coronary atherosclerosis (Stubbs et al., 2016; Zhou et al., 2022). Higher TMAO levels were associated with increased risk of all-cause death in CKD patients from mild to end-stage, which remained significance after controlling for glomerular filtration rates and other covariates, suggesting that TMAO can be an independent predictor for mortality in CKD patients (Missailidis et al., 2016). Similarly, circulating TMAO concentrations were shown to be the highest risk of cardiovascular events in patients with advanced CKD (Kim et al., 2016). Elevated TMAO concentration is associated with renal impairment and dysfunction, which predict poor long-term survival in CKD patients (Tang et al., 2015; Gruppen et al., 2017). However, Kaysen et al. (2015), didn’t find any significant association between serum TMAO concentrations and all-cause mortality, cardiovascular death, or hospitalizations in 235 hemodialysis patients. There were studies that mainly researched 4-5 stage CKD, few about 1–3 stage CKD (Hu et al., 2022). The lack of early-stage CKD patients may limit the application of TMAO as a clinical biomarker of CKD. The level of TMAO in each stage of CKD patients was different (Hu et al., 2022). So the sensibility of TMAO in each CKD patients is unclear. TMAO stands out as a potential biomarker in CKD, which need to validate in the large-scale study and in different populations.

### 2.2 The mechanisms of trimethylamine N-oxide promoting the development of chronic kidney disease

Inflammation is an important mechanism for the TMAO-mediated occurrence and development of CKD. High concentration of TMAO leads to renal interstitial fibrosis and dysfunction by promoting renal oxidative stress and inflammation (Sun et al., 2017). Similarly, a high concentration of TMAO may reduce the production of NO by inducing vascular oxidative stress and inflammation, thereby triggering CKD complications, such as endothelial dysfunction and cardiovascular disease (Li et al., 2018). TMAO activates the NLRP3 inflammasome, leading to the release of IL-1β and IL-18, thereby accelerating kidney inflammation (Fang et al., 2021). Studies have found that TMAO promotes vascular calcification in rats with chronic kidney disease by activating the NLRP3 inflammasome and NF-κB signaling pathway. Antibiotic intervention reduces TMAO levels and reduces vascular calcification (Zhang et al., 2020). In addition, TMAO causes vascular inflammation and myocardial fibrosis to exacerbate cardiovascular disease by activating the NLRP3 inflammasome (Chen et al., 2017; Li et al., 2019a).

The mechanisms by which TMAO may enhance renal damage and aggravate nephropathy have not been well established. TMAO yet activates NF-κB signaling and then induce expression of inflammation gene (Seldin et al., 2016; Yang et al., 2019; Zhang et al., 2019). Besides, activation of NLRP3 inflammasome and NF-κB signaling aggravate kidney disease (Vilaysane et al., 2010; Shahzad et al., 2015). In addition, long-term and excessive consumption of red meat (rich in carnitine and choline) can increase TMAO production and reduce renal excretion (Wang et al., 2019). This indicates that TMAO aggravates inflammation, but the related mechanisms of inducing kidney damage need to be thoroughly investigated. Therefore, it is important to investigate the mechanism of TMAO exacerbating kidney disease through activation of the inflammatory response, which can help TMAO become an effective target.

## 3 Potential treatments or drugs targeting trimethylamine N-oxide

### 3.1 Regulation of gut microbiota

TMAO is related to the abundance of 13 genera of the gut microbiota, including *Prevotella*, *Mitsuokella*, *Fusobacterium*, *Desulfovibrio*, and bacteria belonging to Ruminococcaceae and Lachnospiraceae, as well as the Methane-producing bacteria *Methanobrevibacter smithii* (Fu et al., 2020), which could inspire the development of a drug that specifically targets these gut microbiotas. In other words, drugs that specifically target these gut microbes may have a better TMAO reduction effect. Studies on humans and animals have shown that several bacterial families are involved in the production of TMA-TMAO, such as Deferribacteraceae, Anaeroplasmataceae, Prevotellaceae (Koeth et al., 2013), and Enterobacteriaceae (Craciun and Balskus, 2012; Zhu et al., 2014). Thus, direct regulation of the gut microbiota can serve as one of the targets for regulating the production of TMAO.

#### 3.1.1 Antibiotic

It is considered feasible to reduce TMAO levels by inhibiting the growth of the gut microbiota by using broad-spectrum antibiotics to achieve the purpose of treating chronic kidney disease. Now there is no effective evidence directly demonstrating the feasibility of the treatment, and several large randomized prospective human trials have shown no benefit of antibiotic treatment or prophylactic treatment for CVD. In patients with stable coronary artery disease, taking azithromycin every week for 1 year did not change the risk of cardiac events (Grayston et al., 2005). Long-term use of an antibiotic that is effective against *Clostridium pneumonia* has not been observed to reduce the incidence of cardiovascular events (Cannon et al., 2005). The 3-month course of azithromycin did not significantly reduce the clinical sequelae of coronary heart disease in patients who are in a stable condition with previous myocardial infarction and with Chlamydia pneumonia infection (O’Connor et al., 2003).

Oral antibiotics can inhibit the increase of TMAO level after stimulation of the choline or carnitine diet, suggesting that intestinal bacteria are required for TMAO production (Wang et al., 2011; Koeth et al., 2013; Tang et al., 2013). Discontinuation of antibiotics for 1 month led to the increase of TMAO levels (Tang et al., 2013). This indicates that broad-spectrum antibiotics are not an ideal treatment, because they may have other undesired consequences, and long-term treatment may lead to the emergence of resistant bacteria (Velasquez et al., 2016). CKD patients are often accompanied by gut microbiota dysbiosis, and the use of antibiotics will aggravate the dysbiosis. Although it is unclear whether dysbiosis leads to the increase of TMAO production in CKD patients, it may lead to more serious consequences, such as impaired intestinal mucosal barrier, increased intestinal inflammation (Lange et al., 2016; Vaziri et al., 2016; Velasquez et al., 2016; Andersen et al., 2017). Therefore, antibiotics intervention may be not a desirable method. Targeted TMAO-producing gut microbiota, which undergoes non-lethal inhibition, may be a more reliable method.

#### 3.1.2 Prebiotics and probiotics

Oral prebiotics or probiotics reduce the production of metabolites by changing the composition of the gut microbiota. Regulating the gut microbiota can alter their ability to produce TMA, which may be a reasonable intervention strategy for the prevention or treatment of TMAO-induced metabolic diseases (Kalagi et al., 2019).

*Lactobacillus Plantarum* ZDY04 can significantly reduce the content of TMAO in serum and TMA in the cecum by regulating the structure of gut microbiota community, and effectively inhibit the development of atherosclerosis in mice (Qiu et al., 2018). However, the research on the function of probiotics in the microbial-dependent production of TMA and TMAO is very limited (He and Chen, 2017). So far, there is no data on the potential effects of probiotics on the production of TMAO in humans (Koppe et al., 2015). After mice was administered with probiotic *Lactobacillus paracsei*, the production of TMA was reduced, but TMAO level remained unchanged; but after administered with *Lactobacillus rhamnosus*, the TMAO level was increased, and the TMA concentration in the liver of mice was unchanged (Martin et al., 2008). Treatment with multi-strain probiotic VSL#3 did not appear to reduce the elevated TMAO level in plasma caused by a high-fat diet (Boutagy et al., 2015). After supplementing CKD patients with probiotics (*Streptococcus thermophilus*, *Lactobacillus acidophilus*, and *Bifidobacterium longum*) for 3 months, there was no change in TMAO level, but the betaine level in plasma was significantly increased (Borges et al., 2019). A randomized controlled trial study of probiotics in CKD patients found that probiotics were unable to reduce uremia toxins and inflammation markers (Table 1) (Borges et al., 2018). The efficacy of probiotics in CKD is uncertain, studies in its efficacy are urgently needed.

**TABLE 1:** Probiotic/prebiotic that effect TMAO level.

Probiotic/prebiotic	Result	Mechanism	References
Lactobacillus paracsei	Decreased TMA and invariant TMAO	—	Martin et al. (2008)
Lactobacillus rhamnosus	Increased TMAO and invariant TMA	—	Martin et al. (2008)
Multi-strain probiotic VSL#3	Invariant TMAO in plasma	—	Boutagy et al. (2015)
Lactobacillus plantarum ZDY04	Decreased TMAO in serum and TMA in the cecum	Lactobacillus plantarum ZDY04 regulate structure of the gut microbiota	Qiu et al. (2018)

Coincidentally, the results of various trials in which probiotics were used to alter gut microbiota to improve uremia also show useless (Ranganathan et al., 2010; Miranda Alatriste et al., 2014; Natarajan et al., 2014). The low efficacy of the probiotics limits their clinical application. But Vaziri et al. (2016) proposed that it would be futile to try to restore the required microbiome by introducing beneficial microorganisms without simultaneously improving the biochemical environment of the intestine because the changed structure and function of the gut microbial community are caused by uremic-induced alterations of the intestinal biochemical/biophysical environment. Therefore, improving the gut microbiota environment while using probiotic intervention may enable better efficacy.

Furthermore, future researches need to focus on the interactions between probiotics and TMA production in the gut and activity of flavin monooxygenase (FMO) producing TMAO in the liver also need to be further explored (He and Chen, 2017). Therefore, it is also necessary to consider the impact of probiotic metabolism on the human body. We haven’t understood potential effect of probiotic metabolism *in vivo*.

### 3.2 Target trimethylamine N-oxide generation pathway

#### 3.2.1 Trimethylamine inhibitor

There are four main enzymes involved in the production of TMA: choline-TMA lyase (CutC/D), carnitine monooxygenase (CntA/B), betaine reductase, and TMAO reductase (Barrett and Kwan, 1985; Craciun and Balskus, 2012). In addition, the CntA/B homologous enzyme YeaW/X can also transform betaine, γ-Butyrobetaine, L-carnitine and choline into TMA (Koeth et al., 2014; Iglesias-Carres et al., 2021).

Non-lethal targeting of microorganisms by selectively inhibiting pathways is beneficial to the host. TMA-production related enzymes such as CutC/D, CntA/B, and YeaW/X have become targets of current drug development (Rath et al., 2017). Studies with similar ideas have achieved good results. Structural analogs of choline, 3,3-dimethyl-1-butanol (DMB), which non-lethally inhibits microbial CutC/D reduced TMAO level in mice fed with high choline or L-carnitine (Wang et al., 2015). This research team then modified DMB and found two choline analogs with better effects: iodomethylcholine (IMC) and fluoromethylcholine (FMC) (Roberts et al., 2018). Targetting TMA lyase of gut microbiota is a potential and effective way for the treatment of atherosclerotic heart disease, and inhibiting bacterial CutC is promising to be an effective strategy (Skye et al., 2018). In the isoproterenol-induced CKD mouse model, supplementation of IMC reduced the level of TMA and TMAO by selectively inhibiting the TMA lyase activity of the gut microbiota, consequently ameliorating tubulointerstitial fibrosis (TIF) induced by choline diet, and improving renal function damage. IMC prevents abnormal expression of multiple renal profibrotic genes and reverses multiple changes in gut microbiota composition associated with TMAO and renal function impairment (Gupta et al., 2020). Studies have also found that supplementing IMC can slow down the progression of chronic kidney disease (Zhang et al., 2021) (Table 2).

**TABLE 2:** Target the gut microbiota that produces TMA or its TMA lyase.

Selectively target	Result	Mechanism	References
3,3-dimethyl-1-butanol (DMB)	Decreased TMAO	DMB non-lethally suppresses microbial CutC/D	Wang et al. (2015)
Iodomethylcholine (IMC)	Decreased TMA and TMAO	IMC and FMC selectively target inhibition of choline TMA lyase activity of the gut microbiota	Robert et al. (2018)
Fluoromethylcholine (FMC)			Gupta et al. (2020)
Iodomethylcholine (IMC)	Decreased TMAO level	IMC non-lethal inhibit choline TMA lyase activity	Zhang et al. (2021)

#### 3.2.2 Flavin-containing monooxygenase 3 inhibitor

There are many species of FMO3 substrate. Drugs such as Ranitidine, Benzydamine, Tamoxifen, Sulindac serve as substrates for FMOs (Lawton et al., 1990; Chung et al., 2000). Currently, few types of research reported FMO3 inhibitors, and FMO3 is not easily inhibited (Shimizu et al., 2015). Antisense oligonucleotide knockout FMO3 in mice reduced atherosclerosis, and downregulation of FMO3 not only reduced TMAO level but also regulated lipid metabolism and inflammation, thereby reducing atherosclerosis (Shih et al., 2015). Thiamazole is a classic substrate and competitive inhibitor of FMO3 with high affinity (Schweifer and Barlow, 1996). The 3,3′-Diindolylmethane from Cruciferous vegetables inhibited FMO3 activity and reduces the TMAO level (Chen et al., 2019). Clinical trials demonstrated that consuming indole reduces the relative ratio of TMAO to TMA by inhibiting FMO3 (Cashman et al., 1999), which binds to NADPH-specific residues similar to Thiamazole and competitively suppresses enzymatic activity (Steinke et al., 2020). Obviously, indole isn’t an ideal FMO inhibitor, because of its blood-brain barrier permeability and toxicity of indoxyl sulfate, a liver metabolite of indole (Chen et al., 2016a; Zajdel et al., 2016; Huc et al., 2018) (Table 3).

**TABLE 3:** Compounds that inhibit FMO3 activity.

FMO3 inhibitor	Results	Mechanism	References
Thiamazole	Decreased TMAO level, increased TMA level	Thiamazole is classic substrate and competitive inhibitor of FMO3	Schweifer and Barlow (1996)
3,3′-Diindolylmethane	Decreased TMAO level	3,3′-Diindolylmethane inhibits FMO3	Chen et al. (2019)
			Cashman et al. (1999)
Consuming indole	Decreased the relative ratio of TMAO to TMA	Consuming indole competitively inhibits FMO3	Steinke et al. (2020)

However, regulation of TMAO concentrations by targeting FMO3 remains unfavorable, because the accumulation of TMA will exhibit “Fish Malodor Disorder”, therefore, appropriate targets that regulate TMAO levels need to be investigated (Dolphin et al., 1997; Zhang and Davies, 2016).

### 3.3 Potential drugs

#### 3.3.1 Approved drugs

Ranitidine and Finasteride had potential effects of protecting cardiovascular system and kidneys by improving the composition of the gut microbiota, inhibiting the synthesis and metabolism of TMAO, delaying the deposition of lipids and endotoxins (Liu et al., 2020). Meldonium decreased the gut microbiota-dependent production of TMA/TMAO from L-carnitine in Wistar rats (Velasquez et al., 2016). Meldonium did not influence bacterial growth and bacterial uptake of L-carnitine, but TMA production by the bacteria *K. pneumoniae* was significantly decreased (Kuka et al., 2014). Vitamins B plus vitamin D lowered plasma fasting TMAO. The molecular mechanisms and health implications of these changes are currently unknown (Obeid et al., 2017). Aspirin reduced the level of TMAO by inhibiting the activity of microbial TMA lyase and reducing the atherogenic effects associated with a high-choline diet (SL, 2016), which highlighted the necessity of aspirin as a preventive treatment. In future clinical applications, “prevention before disease onset” enables the efficacy better (Table 4).

**TABLE 4:** Drugs that reduce TMAO level.

Drugs	Results	Mechanism	References
Ranitidine	Decreased TMAO	Ranitidine and Finasteride inhibit the synthesis and metabolism of TMAO by improving composition of the gut microbiota	Liu et al. (2020)
Finasteride			
Meldonium	Decreased the relative ratio of TMA to TMAO	Meldonium doesn’t influence bacterial growth and bacterial uptake of L-carnitine, but TMA production by the gut microbiota bacteria *K. pneumoniae* was significantly decreased	Kuka et al. (2014)
			Velasquez et al. (2016)
Aspirin	Decreased TMAO	Aspirin inhibits the activity of microbial TMA lyase	SL, (2016)
Vitamins B vitamin D	Decreased TMAO in plasma	—	Obeid et al. (2017)

#### 3.3.2 Natural products

**FIGURE 2:**
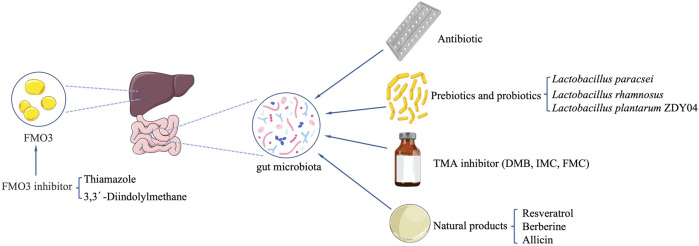
Potential strategies to reduce TMAO formation *in vivo*.

Resveratrol (RSV) attenuated TMAO-induced atherosclerosis (AS) by decreasing TMAO levels and increasing hepatic bile acid (BA) biosynthesis *via* remodeling the gut microbiota (Chen et al., 2016b). Berberine (BBR) attenuated TMA/TMAO production in the C57BL/6J and ApoE KO mice fed with choline-supplemented chow diet and mitigated atherosclerotic lesion areas in ApoE KO mice. BBR altered the gut microbiota composition, function, and cutC/cntA gene abundance. BBR was shown to inhibit choline-to-TMA conversion (Li et al., 2021). *Gynostemma pentaphyllum* (GP) elevated the level of phosphatidylcholine and decreased the level of TMAO (Wang et al., 2013). TMAO is closely related to phosphatidylcholine metabolism. The ability of GP to decrease TMAO levels suggests that GP has an inhibitory effect on the pathway of phosphatidylcholine to TMAO. Allicin reduced TMAO levels in mice fed with high choline. Allicin may be capable of protecting the host from producing TMAO when carnitine is consumed through its impact on the gut microbiota (Wu et al., 2015). *Fructus Ligustri Lucidi* (FLL) reduced FMO3 expression and TMAO level by regulating the gut microbiota in elderly mice (Li et al., 2019b). Consistent intake of Black Raspberry (BR) extract might alleviate hypercholesterolemia and hepatic inflammation induced by excessive choline with a high-fat diet *via* lowering the elevated level of TMA in cecal and TMAO in serum in rats, Polyphenols in BR extract reduced the level of TMA in the cecum by regulating the gut microbiota (Lim et al., 2020). Nobiletin significantly reduced TMAO-induced vascular inflammation via inhibition of the NF-κB/MAPK pathways. Nobiletin decreased TMAO-induced apoptosis of HUVEC cells and counteracted TMAO-induced HUVEC cell proliferation, which indicates that it has a potential ability to reduce TMAO levels (Yang et al., 2019). Black Beans (BB) lowers the abundance of hepatic FMO3, even with a high-fat diet protecting against the production of TMAO, which may be related to modification of the gut microbiota (Sanchez-Tapia et al., 2020) (Table 5; Figure 2).

**TABLE 5:** Natural products that reduce TMAO level.

Natural compounds	Results	Mechanism	References
Resveratrol (RSV)	Decreased TMAO and increased hepatic bile acid (BA)	RSV remodels the gut microbiota	Chen et al. (2016a)
Berberine (BBR)	Decreased the relative ratio of TMA to TMAO	BBR alteres the gut microbiota composition, microbiome functionality, and cutC/cntA gene abundance	Li et al. (2021)
Gynostemma pentaphyllum (GP)	Decreased TMAO and increased phosphatidylcholine	GP has an inhibitory effect on the pathway of phosphatidylcholine to TMAO	Wang et al. (2013)
Allicin	Decreased TMAO	Allicin altered the gut microbiota composition	Wu et al. (2015)
Fructus ligustri lucidi (FLL)	Decreased FMO3 expression and TMAO	FLL regulate the gut microbiota	Li et al. (2019a)
Black raspberry (BR) extract	Decreased TMA in cecal and TMAO in serum	Polyphenols in BR extract enable to reduce the level of TMA in the cecum by regulating the gut microbiota	Lim et al. (2020)
Nobiletin	Nobiletin reduced TMAO-induced vascular inflammation	Nobiletin inhibits of the NF-κB/MAPK pathways	Yang et al. (2019)
Black beans (BB)	Decreased FMO3 expression and TMAO	BB modificate the gut microbiota	Sanchez-Tapia et al. (2020)

Furthermore, the latest research reveals a new mechanism that a high-fat diet promotes the production of TMAO through the gut microbiota. Impaired bioenergetics of mitochondria in the colonic epithelium by high fat diet resulted in the increase of luminal bioavailability of oxygen and nitrate, thereby intensifying respiration-dependent choline catabolism of *E. coli*, which increased the level of circulating TMAO. The 5-aminosalicylic acid (5-ASA) treatment blunts the increase in circulating TMAO in mice on the choline-supplemented high-fat diet (Woongjae Yoo et al., 2021).

Angiotensin-converting enzyme inhibitors (ACEI) and angiotensin receptor blockers (ARBs) are first-line drugs for chronic kidney disease treatment (Excellence, 2021). Their main effect is to protect the kidney and slow the progression of kidney disease by lowering blood pressure and reducing urinary protein. However, ACEI and ARBs are not suitable for all CKD patients (Hricik et al., 1983; Textor et al., 1985; Holm et al., 1996). ACEI is contraindicated in CKD pregnant patients because it is related to neonatal anuria, pulmonary hypoplasia, and neonatal death (Hanssens et al., 1991; Hou, 1999). Therefore, TMAO-targeting drugs are expected to expand the application of patients with CKD, such as probiotics, food-borne natural compounds, etc., whose mild pharmacological effects may be suitable for the vast majority of patients with CKD. In addition, CKD is often associated with a variety of complications, such as mineral and bone disorders (MBD), and it is clinically recommended for regular vitamin D or vitamin D analogs and calcitriol treatment for MBD (Excellence, 2021; Kidney Disease: Improving Global Outcomes, 20112017). Vitamin D has previously been shown to lower TMAO levels (Obeid et al., 2017), it is worthy to investigate the role of Vitamin D in the treatment of CKD and its complications. In conclusion, drugs targeting TMAO are various which have a wide range of sources and great development potential. They are expected to be good complements and alternatives for CKD first-line drugs to overcome the limitations of this drugs, such as the limited applicable population, complicated medications for complications, etc.

## 4 Conclusion and future perspective

TMAO is closely related to chronic kidney disease. It is not only a risk factor for CKD but also a potential biomarker. This review summarizes the currently known drugs, natural compounds, probiotics, and prebiotics that can reduce the level of TMA and TMAO. Targeting the TMA-producing gut microbiota and TMAO-related enzymes are expected to be an important method to reduce TMAO level following the development of research on the gut microbiota and TMAO. It is unlikely that a single biomarker will satisfy the requirement of predicting CKD progression as it would be unlikely to reflect the complexities of the multiple pathophysiological processes involved in CKD progression. Therefore, TMAO may be used as a novel biomarker to assist other biomarkers to improve the accuracy of CKD detection.

Susceptibility to CKD has inter-individual differences (Webster et al., 2017), and difference in TMAO concentration among individuals is an important contributor, which may relate to individual differences in the composition of the microbiota. Genetic variation in the metabolism and disposition of TMAO modifies its concentration, which may be particularly consequential for CKD patients. Due to abnormal human TMA metabolism, the attention on FMO3 SNPs was spurred for many years. Until now more than 300 SNPs of the human FMO3 have been reported (D'Angelo et al., 2013). FMO3 allelic variants have a significant effect on TMAO production, which is closely related to trimethylaminuria (TMAU). The relationship between FMO3 allelic variants and CKD is unclear. FMO3 genotype at amino acid 158 was associated with higher plasma TMAO concentrations, and heterozygous CKD patients (E/K) and homozygous variant CKD patients (K/K) had a greater risk of mortality compared to homozygous CKD patients (E/E) (Robinson-Cohen et al., 2016). Studies had identified that organic cation transporter 2 (OCT2, SLC22A2) mediates TMAO uptake into renal proximal tubular cells and that multiple transporter of the ATP-binding cassette (ABC) family, including ABCG2 (BCRP) and ABCB1 (MDR1), are capable of TMAO efflux (Miyake et al., 2017; Teft et al., 2017). Therefore, gene polymorphisms of OCT2 and ABC in different populations may affect the excretion of TMAO, comprehensive genomic studies of transporters involved in TMAO excretion are required. Studies had found that gut microbiota abundance was significantly altered in CKD and ESRD patients, some of them are TMA producing bacteria, such as *Firmicutes* (Vaziri et al., 2013; Xu et al., 2017; Lun et al., 2019). To consider whether gut microbiota abundance can be used as a biomarker for patients with CKD. However, it is important to note that gut microbiota abundance may vary greatly at different stages of CKD disease, which requires that CKD patients at different stages are classified and studied in larger human cohorts.

In conclusion, there are many targets that regulate the production of TMAO. Nonlethal inhibition of the gut microbiota CutC/D, CntA/B et al. are of more clinical significance. Further research is needed to develop probiotics and probiotics to make them work *in vivo*. Researching approved drugs that can reduce TMAO levels with the help of the idea of “repurposing” would be able to save a lot of money for the early development of drugs. Natural compounds are abundant which require extensive screening and investigating the mechanisms. Besides, there are other methods to reduce the TMAO levels, such as taking advantage of the physical properties of the drug, AST-120 is a carbon preparation (Brand name: KREMEZIN), its efficient adsorption function can attenuate absorption of hazardous metabolites *in vivo*. The use of AST-120 in animals and patients with chronic kidney disease can reduce oxidative stress and inflammation, and slow down the progression of kidney disease (Shibahara and Shibahara, 2010; Nakamura et al., 2011; Bolati et al., 2012; Ito et al., 2013). There are also other potential inhibitors, It was reported that many choline-utilizing gut microorganisms can hydrolyze phosphatidylcholine (PC) using a phospholipase D (PLD) enzyme and further convert the released choline to TMA (Chittim et al., 2019), Thus searching for inhibitors that target PLD without affecting host enzymes may reduce PC hydrolyze, then reducing TMAO production.
